# Optimization of fluidized bed drying of *Lactiplantibacillus plantarum* grown in sustainable culture media for winemaking application

**DOI:** 10.3389/fmicb.2025.1695290

**Published:** 2025-11-06

**Authors:** Marina E. Navarro, Manuel A. Morales, Natalia S. Brizuela, Adriana C. Caballero, Andrés Reyes-Urrutia, Fausto Vicente, Liliana C. Semorile, Bárbara M. Bravo-Ferrada, Emma E. Tymczyszyn

**Affiliations:** 1 Laboratorio de Microbiología Molecular, Instituto de Microbiología Básica y Aplicada (IMBA), Universidad Nacional de Quilmes (UNQ), Comisión de Investigaciones Científicas de la Provincia de Buenos Aires (CICPBA), Bernal, Argentina; 2 Instituto de Investigación y Desarrollo en Ingeniería de Procesos, Biotecnología y Energías Alternativas (PROBIEN)- Consejo Nacional de Investigaciones Científicas y Técnicas (CONICET)- Universidad Nacional del Comahue (UNCo), Neuquén, Argentina; 3 Facultad de Ciencias y Tecnología de los Alimentos (FACTA)- Universidad Nacional del Comahue (UNCo), Villa Regina, Río Negro, Argentina; 4 Facultad de Ingeniería (FI)-Universidad Nacional del Comahue (UNCo), Neuquén, Argentina

**Keywords:** *Lactiplantibacillus plantarum*, apple pomace, whey permeate, malolactic fermentation, fluidized bed drying, preservation

## Abstract

Lactic acid bacteria (LAB) play a key role in winemaking by driving malolactic fermentation, which enhances microbial stability and reduces wine acidity. To enable the large-scale development of LAB starter cultures, cost-effective biomass production and preservation methods are required. Traditional preservation techniques, such as freeze-drying, can be expensive and energy-intensive. Therefore, this study explored fluidized bed drying as a sustainable and low-cost alternative for preserving *Lactiplantibacillus plantarum* UNQLp 11 cultured in apple pomace (AP) and whey permeate (WP)-based media, with emphasis on maintaining malolactic activity after preservation. *L. plantarum* UNQLp 11 was cultivated in AP- and WP-based media and subjected to fluidized bed drying at 45 °C and 60 °C. Drying times and culture conditions (pH, medium composition) were varied to assess their impact on cell viability. After drying, the samples were stored for 12 months at 4 °C to evaluate long-term stability. Water activity (aw) was monitored and adjusted between 0.10 and 0.33. Malolactic activity was tested through winemaking trials using synthetic and Malbec wines, both with and without rehydration in DeMan-Rogosa-Sharpe (MRS) broth. Fluidized bed drying caused viability reductions of 3–5 log units, with survival rates influenced by medium composition, pH, and drying duration. Optimal results were achieved at 45 °C for 35 min, yielding final counts of 8.3 log CFU/g in WP cultures and 7.0–7.4 log CFU/g in AP cultures. After 12 months of storage at 4 °C, viability losses were limited to approximately 1 log unit when aw values were maintained between 0.10 and 0.33. In winemaking assays, WP-derived cultures preserved malolactic activity, consuming 70–100 % of malic acid in synthetic wine and up to 80 % in Malbec wine. AP cultures required rehydration to restore performance, reaching 75–100 % and 50–80 % malic acid consumption in synthetic and Malbec wines, respectively. The results demonstrate that fluidized bed drying is a viable and energy-efficient alternative to freeze-drying for preserving *L. plantarum* UNQLp 11. Cultures produced in WP medium exhibited superior viability and maintained malolactic functionality after long-term storage. However, AP-based cultures required rehydration to regain full activity. These findings highlight the potential of fluidized bed drying for sustainable LAB preservation in winemaking applications, although further optimization of drying parameters and protective agents is needed to enhance industrial applicability.

## 1 Introduction

The development of efficient, low-cost, and sustainable strategies for the preservation of malolactic starter cultures is a central challenge in modern winemaking. These cultures are essential to ensure reliable malolactic fermentation (MLF). Malolactic starter cultures consist of selected lactic acid bacteria (LAB) strains, mainly *Oenococcus oeni* and *Lactiplantibacillus plantarum*, with specific properties that ensure the development of a successful and safe fermentation process (Fu et al., 2022; Paramithiotis et al., 2022; Payan et al., 2023). Although LAB biomass can be produced using commercial culture media, the high costs of these media make the scalability of this process economically unfeasible. Therefore, the formulation of low-cost media that can achieve similar bacterial yields while preserving enological and technological properties would be highly valuable for the wine industry, offering a cost-effective alternative for large-scale production. In this context, the use of by-products from the food industry presents an excellent alternative and helps address the environmental issues associated with their disposal (Bravo et al., 2019). In this study, two alternative culture media that had been previously studied in our laboratory were used. These media were formulated with apple pomace (AP), a residue from the non-concentrated juice and cider industry rich in simple sugars such as glucose, fructose, and sucrose (Bravo et al., 2019), and whey permeate (WP), a by-product of cheese and other dairy products, obtained through the filtration of whey, which is rich in lactose, minerals, and vitamins (Brizuela et al., 2021). Supplementation of AP and WP with yeast extract, salts (Anedra, Buenos Aires Argentina), and Tween 80 yields *Lactiplantibacillus plantarum* growth results comparable to those obtained with De Man–Rogosa–Sharpe (MRS) broth, which is the commercial medium used for LAB cultivation (Brizuela et al., 2021; Cerdeira et al., 2021).

The industrial application of LAB starter cultures largely depends on the preservation technologies used, which are necessary to ensure the long-term delivery of stable cultures in terms of viability and activity (Santivarangkna et al., 2008). The technologies most commonly used for LAB preservation are freezing and freeze-drying (Meena et al., 2023). However, due to their high cost and time-consuming nature, alternative methods such as spray drying, fluidized bed drying, and vacuum drying have been explored in recent years as the cost-effective options for large-scale powder production. These technologies, which are based on cellular dehydration, expose cells to thermal, mechanical, osmotic, and oxidative stress (Gagneten et al., 2024; Mari et al., 2024). To minimize damage, protective agents (e.g., sugars, oligosaccharides, complex polymers, and starch) are added to cell concentrates or used as dehydration media (carriers) to stabilize the cultures (Türker et al., 2006). Fluidized bed drying has been traditionally used for yeast preservation (Mille et al., 2004; Soltani et al., 2020), but this method has been poorly explored for bacterial dehydration (Stummer et al., 2012). In addition to the low investment and maintenance costs of the equipment for large-scale production, fluidized bed dryers offer advantages over other drying methods, as they provide efficient mixing of solids and large interfacial areas. This leads to relatively high heat and mass transfer rates, resulting in more homogeneous drying and a significant reduction in dehydration temperatures and times (Fan et al., 2008; Bravo-Ferrada et al., 2013; Reyes Urrutia et al., 2016; Soltani et al., 2020. Fluidized bed drying technology requires the material to be dried in pellet form. Therefore, the biomass must be entrapped, embedded, or encapsulated within a support material (Türker et al., 2006), some of which, depending on their chemical composition, may also serve as protective agents against dehydration.

This study aimed to explore the preservation of a LAB strain native to the Río Negro Province of Argentina, *Lactiplantibacillus plantarum* UNQLp 11, which was isolated from Pinot noir wine (Bravo-Ferrada et al., 2013; Brizuela et al., 2017, 2018). To achieve this objective, UNQLp 11 was cultured in AP- and WP-based media, mixed with manioc starch, and dehydrated by fluidized bed drying at 45 °C and 60 °C. Storage stability, survival, and malolactic consumption capacity in synthetic and Malbec wines were analyzed to elucidate the best preservation conditions in order to ensure a long shelf life while maintaining its technological and enological properties.

## 2 Materials and methods

### 2.1 Bacterial strain

*Lactiplantibacillus plantarum* UNQLp 11 (GeneBank Accession Number: CP031140) was isolated from Patagonian Pinot Noir red wine (Brizuela et al., 2017; Iglesias et al., 2020), in which the MLF was spontaneous. It is deposited in the strain collection of the Laboratorio de Microbiología Molecular (Universidad Nacional de Quilmes, Bernal, Buenos Aires, Argentina). Cultures were kept frozen at −20 °C in MRS broth (Biokar Diagnostics, Beauvais, France) (De Man et al., 1960), with glycerol (20% v/v). Cells were grown in 10 mL of MRS broth at 28 °C for 48 h in aerobic conditions.

### 2.2 Growth in apple pomace-based culture medium

Apple pomace (AP), a free additive by-product from the production of apple juices, was generously donated by Patagonia Beverage SRL (Province of Río Negro, Argentina) and was physicochemically characterized in a previous study (Navarro et al., 2024). Both AP and AP with 50% w/v distilled water dilution (APW) were supplemented with yeast extract (Britania, Buenos Aires, Argentina) 1% w/v, Tween 80 (Biopack, Buenos Aires, Argentina) 1 mL/L, cysteine HCl 0.5 g/L, NH4-citrate 2 g/L, K_2_HPO_4_ 2 g/L, MgSO_4_ 0.1 g/L, and MnSO_4_ 0.05 g/L, as described by Cerdeira et al. (2021). The pH of the media was adjusted to 4.5 and 6.0 and sterilized in an autoclave at 121 °C for 15 min. Then, 120 g of these culture media were inoculated with UNQLp 11 at 1% v/w (initial inoculum of 5 × 10^7^ CFU/g) and incubated at 28 °C for 48 h in aerobic conditions without agitation (Navarro et al., 2024), until it reached the stationary phase (~1 × 10^10^ CFU/g).

### 2.3 Growth in whey permeate-based culture medium

Whey permeate (WP), a by-product obtained by drying deproteinized sweet whey containing approximately 80% w/w lactose, 6% w/w ashes, and 3% w/w proteins, was generously supplied by Arla Foods Ingredients S.A. (Buenos Aires, Argentina). The WP-based culture medium used here was prepared by adding the following components to a dry whey solution at a concentration of 5% w/v: yeast extract (Britania, Buenos Aires, Argentina) 0.5% w/v, peptone 0.5% w/v, NH4-citrate 2.0 g/L, K_2_HPO_4_ 2 g/L, Tween 80 (Biopack, Buenos Aires, Argentina) 1 mL/L, MgSO_4_ 0.1 g/L, and MnSO_4_ 0.05 g/L (Cerdeira et al., 2019). The pH of the medium was adjusted to 4.5 and 6.0, and the medium was sterilized in an autoclave at 121 °C for 15 min. Then, 5 L of WP-based medium was inoculated with UNQLp 11 at 1% v/v (2 × 10^7^ CFU/mL) and incubated at 28 °C for 48 h in aerobic conditions without agitation (Brizuela et al., 2021), until it reached the stationary phase (~4 × 10^10^ CFU/mL).

### 2.4 Fluidized bed drying

**Figure 1:**
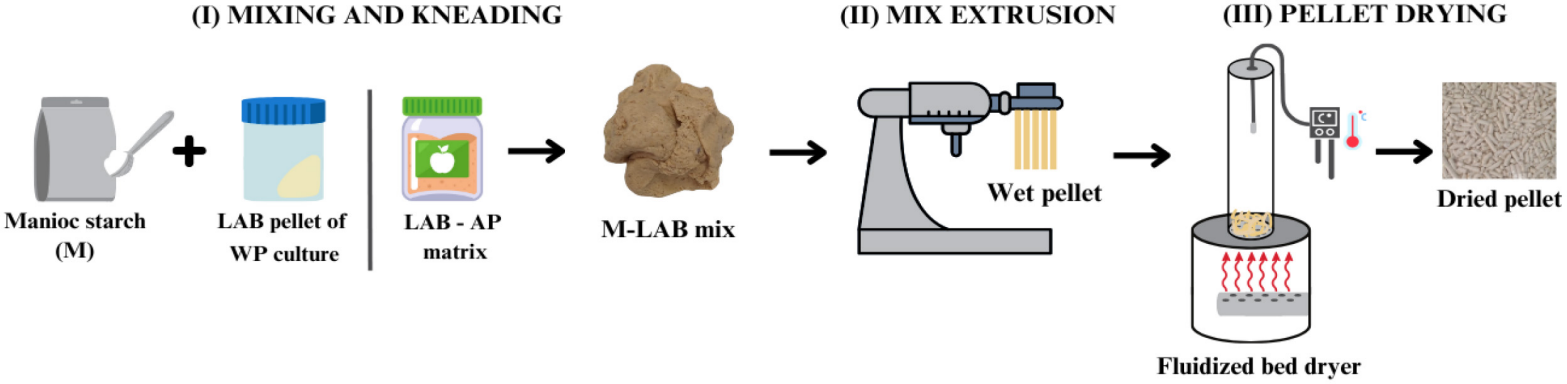
Lactic acid bacteria (LAB) fluidized bed drying process. (I) Mixing and kneading of the LAB pellet after centrifugation of the WP culture or LAB in the AP matrix with manioc starch. (II) Mix extrusion with a laboratory extruder machine. (III) Wet pellet drying in a fluidized bed dryer.

The bacterial biomass, recovered by centrifugation from WP-based media and cultured in AP-based medium, was weighed and mixed at an approximately 1:1 ratio with manioc starch (until a dough consistency is achieved with good kneading characteristics) and an anticaking agent. Then, to obtain a wet pellet, the mix was extruded with a 2-mm pore screen plate with the laboratory extruder machine (PEABODY-Model: PE-MP001R). This procedure is illustrated in Figure 1.

Wet pellets were dehydrated in the fluidized bed dryer at 45 and 60 °C. The fluidized bed dryer consists of a cylindrical column with a 0.0533-m diameter and 1-m height. Air, used as a fluidizing agent, was supplied by a blower, and particle suspension was achieved through an air distributor located at the bottom of the bed. The operating temperature and air velocity were controlled by an automatic proportional–integral–derivative controller and a manual valve, respectively. The average air velocity within the bed was 3.074 m/s. Additionally, the relative humidity of the air at the outlet of the bed was monitored using a humidity sensor (Morales et al., 2025).

The experiment began by loading 50 g of wet pellets into the bed from the top. The samples were taken at the beginning (*t* = 0 min) and thoroughly processed every 10 or 15 min.

### 2.5 Water activity and moisture content determination

The water activity (a_w_) of wet and fluidized bed dried samples was measured using AquaLab—Model Series 3 TE instrument (Decagon Devices, Inc., WA, USA).

The moisture content (MC) of dehydrated pellet samples is expressed as a percentage of wet weight (MC%). Dehydrated pellet samples were desiccated in an oven at 105 °C until constant weight, and moisture content was calculated following Equation 1, where M1 and M2 are the weights before and after drying, respectively.


$$\begin{array}{l} MC\%=\frac{\left(M1-M2\right)}{M1}\;\times \;100 \end{array}$$ (1)

The Guggenheim Anderson-de Boer [GAB] mathematical model (Guggenheim, 1966; Anderson, 1946; De Boer, 1953) was used to describe the relationship between moisture content and water activity. Isotherm adjustment was performed according to Equation 5 (see results).

### 2.6 Storage conditions

Dehydrated samples were stored at 4 °C in a vacuum-sealed package (vacuum packaging machine FoodSaver^®^ OSTER tumble dryer — model V204), protected from light.

### 2.7 Winemaking trials in synthetic wine

Samples with the highest viability from each alternative culture medium were selected for vinification trials in synthetic wine. Dried samples were rehydrated at a 1:2 w/v ratio using physiological solution (NaCl 0.9% w/v) at 25 °C for 15 min. The samples grown in AP-based media were additionally rehydrated in MRS broth at 25 °C for 15 min. Following rehydration, the samples were centrifuged and inoculated into synthetic wine at a 1:10 ratio (1 g of pellet in 10 mL of synthetic wine). The composition of synthetic wine was tartaric acid 5 g/L, L-malic acid 2.3 g/L, acetic acid 0.6 g/L, glucose 2 g/L, fructose 2 g/L, and ethanol 12.0% v/v, pH 3.7. Cell viability and L-malic acid consumption were analyzed before and after 7 days of fermentation at 21 °C.

### 2.8 Winemaking trials in Malbec wine

Based on the results in synthetic wine, samples with the highest final percentage of malic acid consumption were selected for trials in Malbec wine. Dried LAB were rehydrated at a 1:2 w/v ratio using physiological solution (NaCl 0.9% w/v) (LAB grown in WP-based medium) or in MRS broth (LAB grown in AP-based media) at 25 °C for 15 min. Following rehydration, the samples were centrifuged and inoculated into Malbec wine at the final stage of alcoholic fermentation (pH 3.74, ethanol 14.5% v/v, and L-malic acid 1.6 g/L) at a 1:10 ratio (1 g of pellet in 10 mL of Malbec wine). Growth in MRS broth without treatment was used as a positive control, and wine without inoculation was used as a negative control.

Cell viability and malolactic activity were analyzed on days 0, 2, 4, 5, 7, 12, and 20 of fermentation, with incubation carried out at 21 °C.

### 2.9 Cell viability

Cell viability was determined before and after dehydration, after 1 year of storage, and during the fermentation process in synthetic and Malbec wines.

Before and after dehydration, as well as after 1 year of storage, the samples were rehydrated 1:2 w/v with physiological solution (NaCl 0.9% w/v) at 25 °C for at least 15 min. Cell viability was determined by bacterial colony count on MRS plates. Serial dilutions (1:10) with 0.9% w/v NaCl solution were carried out, and the plates were incubated at 28 °C for 48 h, in aerobiosis. The results are expressed as log CFU/g.

In samples from synthetic wine, the count was realized on MRS agar plates. In samples from Malbec wine, the count was performed in MRS agar added with 100 μL of sodium azide and cycloheximide 1% (w/v) to prevent the growth of mold and yeast.

### 2.10 Malolactic activity

The L-malic acid remaining in the fermented wine samples was measured with an L-malic acid enzymatic kit (L-Malic Acid Enology enzymatic kit, Bio Systems SA, Barcelona, Spain). The percentage of L-malic acid consumption (%MAC) was obtained using Equation 2,


$$\begin{array}{l} \%MAC=100-\frac{\left[MA_{t}\right]100}{\left[MA_{0}\right]} \end{array}$$ (2)

where [*MA*_*t*_] is the L-malic acid concentration at time = *t* and [*MA*_0_] is the initial concentration of L-malic acid. At the same time, an exponential one-phase decay equation model was used to fit the MAC kinetics of the different strains tested in wine, by using Equation 3, obtained by GraphPadPrism^®^ 6.01 software (Graph Pad Software, Inc., San Diego, CA, USA, 2007),


$$\begin{array}{l} \%MACt=\left(\%MACi\right)\left(1-\;e^{-kt}\right) \end{array}$$ (3)

where %*MAC*_*t*_ is the L-malic acid consumed at time *t*, %*MAC*_*i*_ is the maximum L-malic acid consumed at infinite time (plateau), and *k* is the rate constant.

### 2.11 Statistical analysis

All experiments were carried out in duplicate. The results presented correspond to the median ± standard deviation. Analysis of variance (ANOVA) was carried out using statistical software STATISTIX 8 (Analytical Software, Tallahassee, FL, USA). Means were compared using Tukey's test for multiple comparisons, and the difference was considered significant when *p*-value was < 0.05.

## 3 Results

### 3.1 Kinetics of fluidized bed drying of sustainable LAB cultures

**Figure 2:**
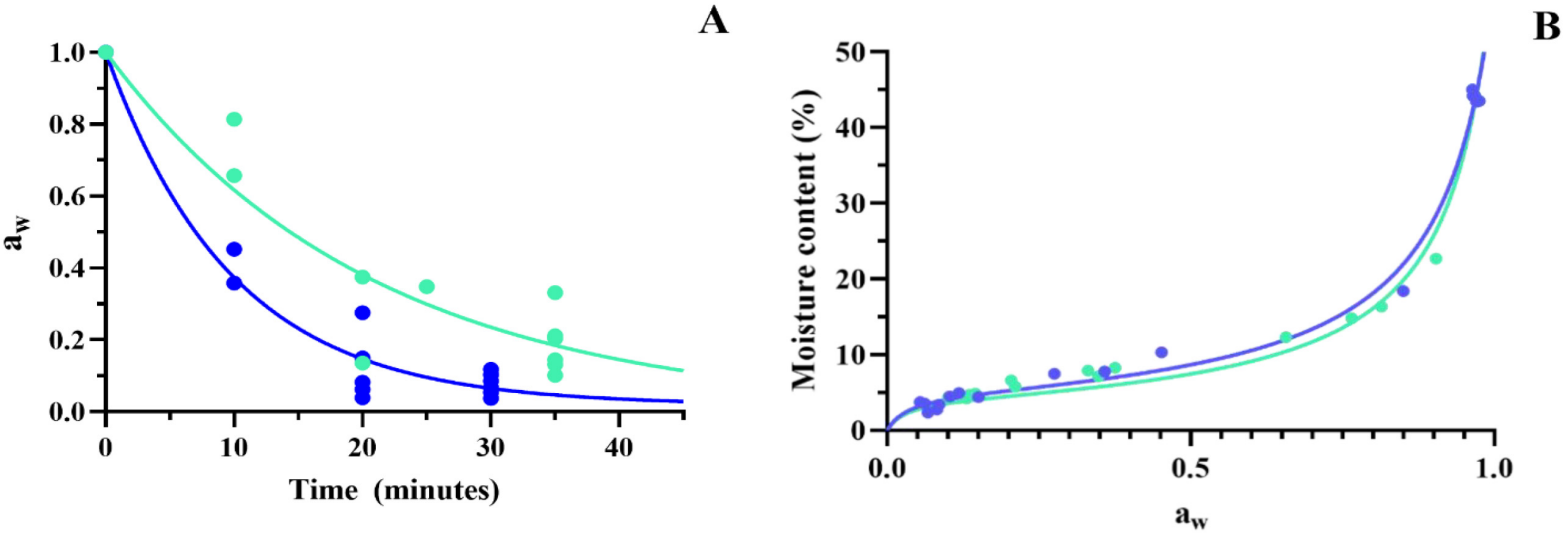
Kinetics of fluidized bed drying of *Lpb. plantarum* UNQLp 11 grown in alternative media. **(A)** Exponential decay of water activity (a_w_) through the drying process and **(B)** desorption isotherms of the biomass dried at 45 °C (green line) and 60 °C (blue line).

Figure 2A shows the a_w_ exponential decay function (Equation 4), where *k* is the rate constant and *t* is the time of dehydration, for both temperatures. As the rate constant at 60 °C (0.1) was twice the rate constant at 45 °C (0.05) (Table 1), then, the a_w_ decay kinetics, that is, the dehydration rate, was faster at 60 °C than at 45 °C, achieving the a_w_ values below 0,20 and 0,40 at 60 and 45 °C, respectively. A high dispersion of a_w_ value is observed over time, and this may be due to differences in the initial water content of samples previous to drying.


$$\begin{array}{l} a_{w}=\;e^{-kt} \end{array}$$ (4)

**Table 1:** Exponential decay of water activity at 45 and 60 °C fluidized bed dehydration treatment of *Lpb. plantarum* UNQLp 11 grown in alternative media.

**UNQLp 11 dehydration**		
**Temperature (**°**C)**	***k***	*R* ^2^
45	0.05	0.86
60	0.10	0.94

*k*, constant of first-order exponential decay; *R*^2^, coefficient of determination.

Figure 2B shows the desorption isotherms of the bacterial dried samples, representing the relation between moisture content and a_w_ at both dehydration temperatures.

Isotherms were adjusted using the GAB model (Equation 5) (Anderson, 1946; De Boer, 1953; Guggenheim, 1966), where *Mo* is the monolayer moisture content, *G* is a constant related to the first layer heat of sorption, *k* is a constant related to the multilayer heat of sorption, and *m* represents the moisture content of the sample.


$$\begin{array}{l} a_{w}=\frac{\frac{G.Mo}{m}-\left(G-2\right)-\sqrt{{\left(G-\;\frac{G.Mo}{m}\right)}^{2}+\;\frac{4G.\;Mo}{m}}}{2\;\left(1-G\right)k} \end{array}$$ (5)

Both temperature curves, 45 and 60 °C, exhibited a good fit to the GAB model, as indicated by the determination coefficient (*R*^2^) of 0.99 (Table 2). This high correlation demonstrates that the model accurately describes the relationship between equilibrium moisture content and a_w_ for the samples analyzed.

**Table 2:** Constant values obtained from the Guggenheim Anderson-de Boer (GAB) model equation fit in the desorption isotherm of *Lpb. plantarum* UNQLp 11 cultures fluidized bed dried at 45 and 60 °C.

**Temperature (°C)**	**Mo**	**G**	***k***	***R* ^2^**
45	4.10	36.00	0.94	0.99
60	4.86	32.13	0.92	0.99

Mo, monolayer moisture content; G, constant related to the heat of sorption of the first layer; *k*, constant related to the heat of sorption of the multilayer; *R*^2^, coefficient of determination.

### 3.2 Cell survival of dehydrated LAB cultures

**Figure 3:**
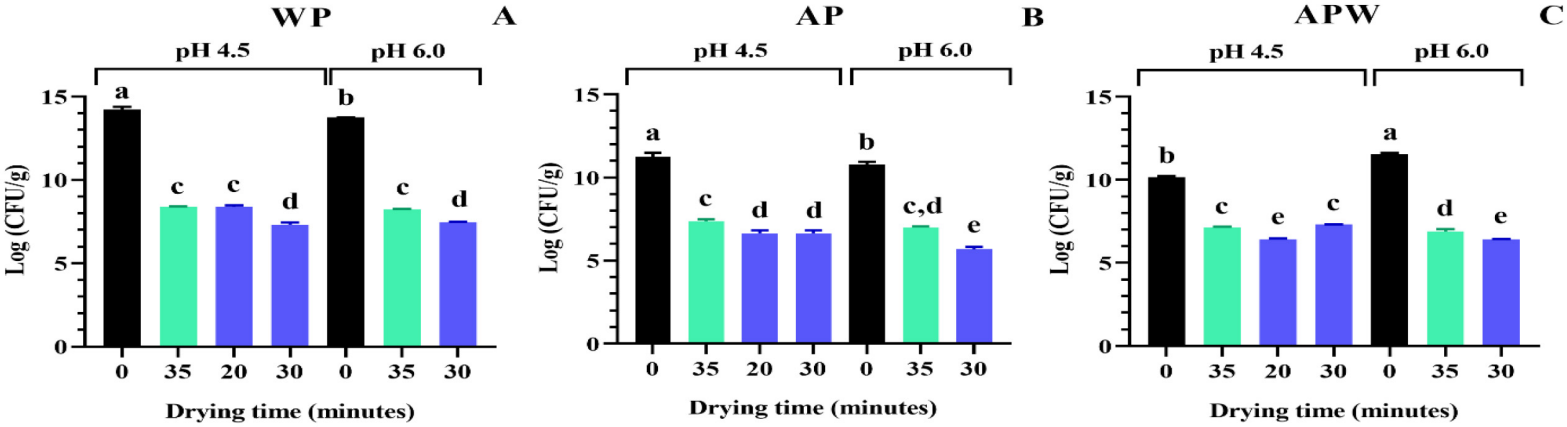
Viability of *Lpb. plantarum* UNQLp 11 grown in **(A)** whey permeate-based media (WP), **(B)** apple pomace-based media (AP), and **(C)** apple pomace plus water-based media (APW), before (black) and after fluidized bed drying at 45 °C (green) and 60 °C (blue). The different letters (a–e) represent groups with significant differences (*p* > 0.05) according to the ANOVA and Tukey's test statistical analysis.

The cell viability of *Lpb. plantarum* UNQLp 11 was evaluated before and after fluidized bed drying during different times at 45 and 60 °C, as well as after 1 year of storage. Figure 3 shows cell viability after 48 h of the fluidized bed drying process for different conditions of cultures and dehydration times.

Figure 3A shows the viability of UNQLp 11 cultured in WP-based medium, where the initial cell count was ~1 × 10^14^ CFU/g of a wet pellet) (Figures 3B, C), as LAB cells in this WP liquid culture were concentrated by centrifugation prior to the mixing step. These samples were severely damaged after drying, as viability was observed to decrease up to 5 log units, with no significant differences between the two temperatures. This figure also shows that the highest viability value after drying (8.3 log CFU/g) was achieved when the culture grown at pH 4.5 was dried at 45 °C and 60 °C for 35 and 20 min, respectively, as well as when grown at pH 6.0 and dried at 45 °C for 35 min.

The AP-based medium had a lower initial cell count (~1 × 10^10^ CFU/g of wet sample) than the WP-based medium, as bacteria grown in these semi-solid culture media were not concentrated, by centrifugation, before dehydration (Figures 3B, C). The AP-based medium (Figure 3B) showed a decrease in cell viability between 3 and 5 log CFU/g, with the highest viability values (7.0–7.4 log CFU/g) recorded at 45 °C for 35 min for the cultures grown at both pHs, and at 60 °C for 20 min for the culture grown at pH 4.5. In contrast, the APW medium (Figure 3C) showed the highest viability values (6.9–7.3 log CFU/g) recorded at 45 °C for 35 min for the cultures grown at both pHs, and at 60 °C for 30 min of drying for those grown at pH 4.5.

The optimal drying condition for UNQLp 11 grown in WP and AP-based media was generally mostly achieved at 45 °C for 35 min of drying, regardless of the pHs.

**Figure 4:**
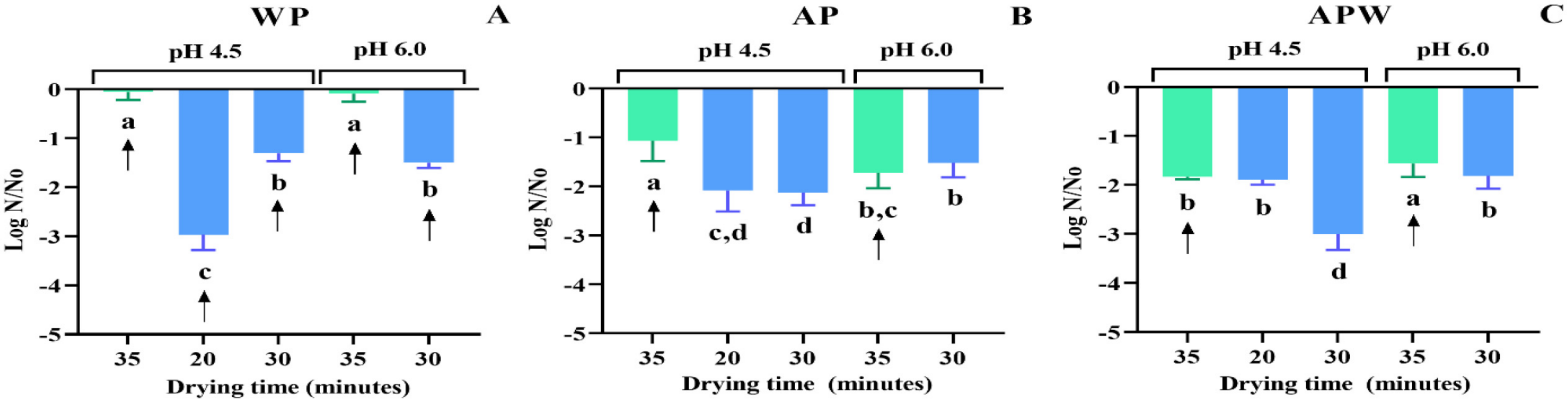
Viability loss of *Lpb. plantarum* UNQLp 11 grown **(A)** whey permeate-based media (WP), **(B)** apple pomace-based media (AP), and **(C)** apple pomace plus water-based media (APW), fluidized bed dried at 45 °C (green) and 60 °C (blue) after 1 year of storage. The different letters (a–d) represent groups with significant differences (*p* > 0.05) according to the ANOVA and Tukey's test statistical analysis. Samples marked with arrows showed the highest survival rates and were selected for subsequent winemaking trials in synthetic wine.

Next, to analyze storage effects, dried samples were vacuum-packed and stored without light exposure for 12 months at 4 °C. After this time, the viability of dehydrated UNQLp 11 was evaluated. Results were variable according to the different pH values and drying times (Figure 4). Figure 4A shows that, for the samples grown in WP-based medium, the optimal preservation condition after 1 year was drying at 45 °C for 35 min to maintain cell viability (for culture pH of 4.5 and 6.0), without significant differences regarding samples immediately after drying, which showed values of 8.4 and 8.3 log CFU/g for pH values of 4.5 and 6.0, respectively (Figure 3A).

Figures 4B, C show that cell viability of the pellet samples grown in AP-based media at pH 4.5 (stored under the same conditions), the lowest viability loss (1 and 1,5 log CFU/g, respectively, compared to those without storage) was observed in the pellets dried at 45 °C for 35 min. However, the AP and APW pellet samples grown at pH 6.0, regardless of the drying temperature, show the same viability at their higher drying time (Figures 4B, C).

**Figure 5:**
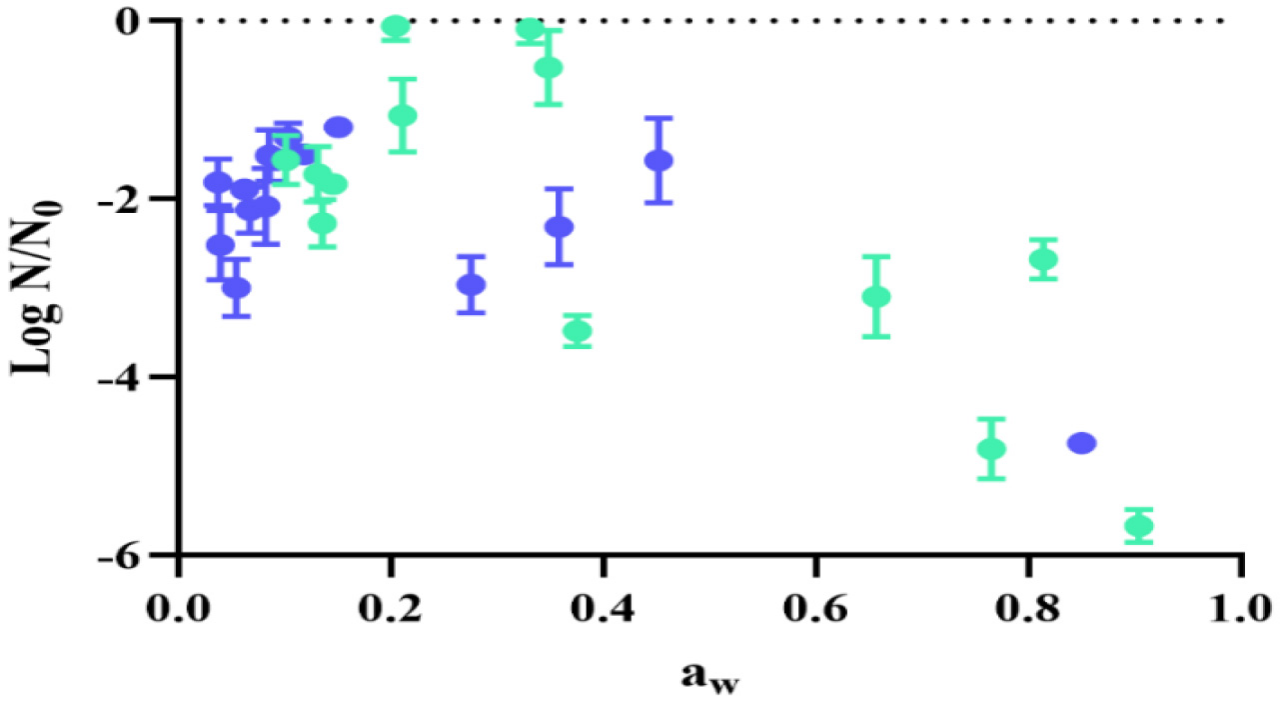
Viability loss of *Lpb. plantarum* UNQLp 11 grown in alternative culture media, fluidized bed dried at 45 °C (green) and 60 °C (blue) after 1 year of storage, in function of the samples' water activity (a_w_).

Figure 5 shows the viability loss (log N/N_0_) of UNQLp 11 after 12 months of storage, as a function of a_w_ after fluidized bed drying at both temperatures, independently of the growth conditions. This figure also shows that viability maintenance is dependent on the final a_w_ of samples, being the optimal a_w_ range between 0.10 and 0.33. Outside this range, a greater loss of viability is observed, particularly at higher a_w_ (corresponding to lower times of drying).

### 3.3 Winemaking trials with fluidized bed dehydrated UNQLp 11 biomass

The malic acid decarboxylation capacity and cell survival in wine stress conditions of the dried culture stored for 12 months were analyzed in synthetic wine and Malbec wine at the final stage of alcoholic fermentation. The dehydrated and stored samples that showed the highest survival rates were selected for vinification trials in synthetic wine (arrows shown in Figure 4).

The dried WP culture samples with high viability, selected after 1 year of storage, were rehydrated with physiological solution. Results showed that they retained their malolactic activity with a final malic acid consumption ranging from 70 to 100% (Table 3). In contrast, dried AP culture samples with high viability rehydrated with the same solution showed low malic acid consumption throughout the week of incubation, ranging from 18 to 40%. Samples rehydrated with MRS broth showed malic acid consumption values between 75 and 100% (Table 4).

**Table 3:** Percentage of L-malic acid consumption (%MAC) after 7 days of fermentation, in synthetic wine at 21 °C, of fluidized bed dried *Lpb. plantarum* UNQLp 11 grown in whey permeate (WP) based media after 1 year of storage.

**Final %MAC in synthetic wine fermentation for WP culture samples**			
**Temperature (**°**C)**	**Time (min)**	**pH**	**WP %MAC**
45	35	4.5	100 ± 3.22^*^
		6.0	76.04 ± 1.90
60	20	4.5	80.74 ±1.85
	30	4.5	79.87 ± 1.44
		6.0	86.62 ± 1.77^*^

^*^Conditions selected for winemaking in Malbec wine.

**Table 4:** Percentage of L-malic acid consumption (%MAC) after 7 days of fermentation, in synthetic wine at 21 °C, of fluidized bed dried *Lpb. plantarum* UNQLp 11 grown in apple pomace (AP) based media after 1 year of storage.

**Final %MAC in synthetic wine fermentation for AP and APW culture samples**						
**Temperature (**°**C)**	**Time (min)**	**pH**	**AP**	**%MAC**	**APW %MAC**	
			**PS**	**MRS**	**PS**	**MRS**
45	35	4.5	41.69 ± 6.77	80.45± 8.88^*^	18.69 ± 6.28	87.01 ± 13.61^*^
		6.0	28.35 ± 2.65	76.46 ± 4.06^*^	36.82 ± 7.99	98.48 ± 3.46^*^

Samples were rehydrated before the fermentation process with physiological solution (PS) or MRS broth.

^*^Conditions selected for winemaking in Malbec wine.

**Figure 6:**
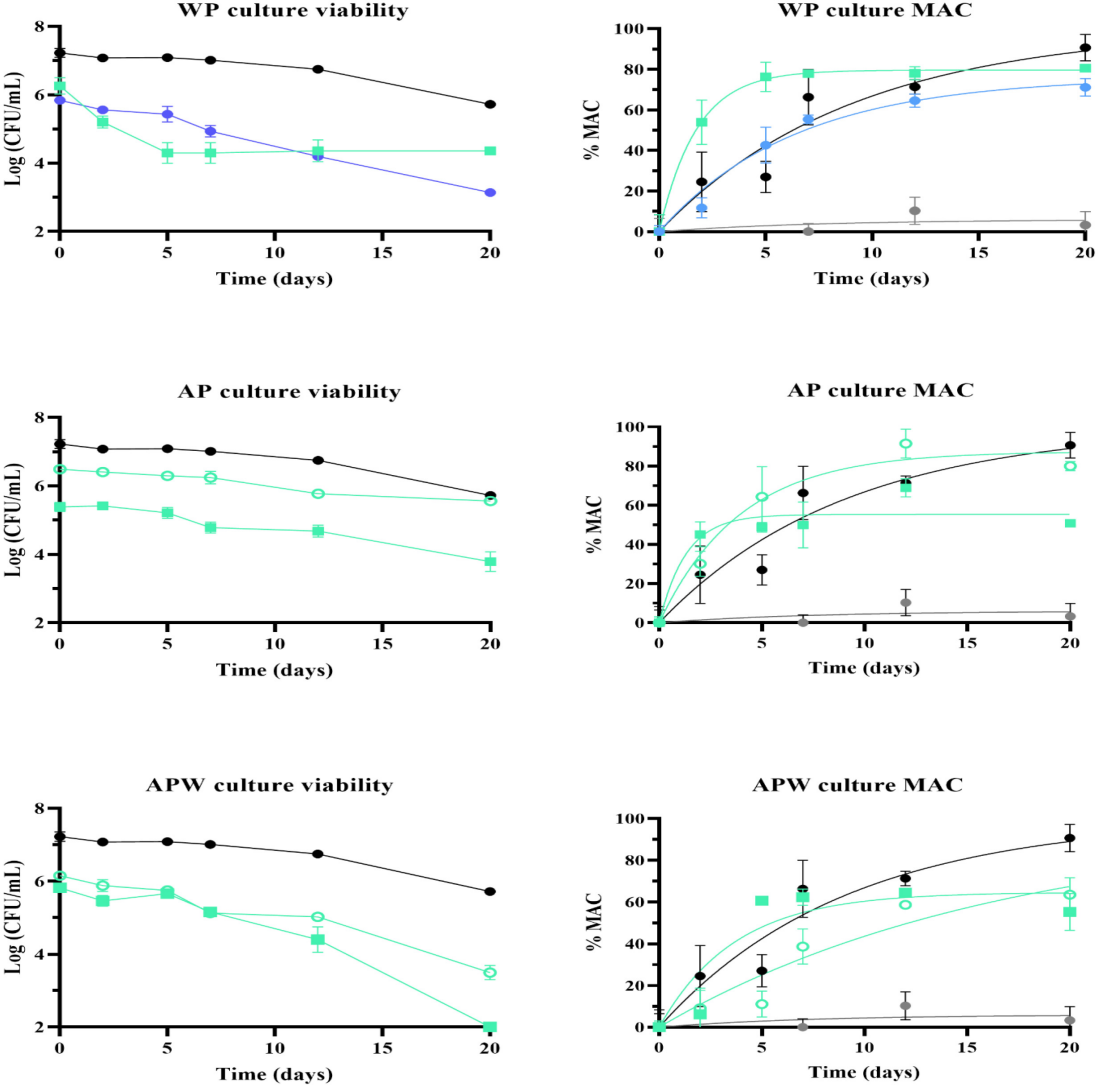
Cell viability (left) and percentage of malic acid consumption (right) in Malbec wine (pH 3.74 and 14.5% v/v ethanol), incubated at 21 °C for 20 days, of *Lpb. plantarum* UNQLp 11 grown in **(A)** whey permeate-based media (WP), **(B)** apple pomace-based media (AP) and **(C)** apple pomace plus water-based media (APW), with initial pH of 4.5 (squares) and 6.0 (dots), and dehydrated by fluidized bed drying at 45 °C for 35 min (green) and 60 °C for 30 min (blue). UNQLp 11 grown in MRS broth without a preservation process applied was used as a positive control (black), and wine without inoculation as a negative control (gray).

Based on the results of the vinification trials in synthetic wine, samples with the highest final percentage of malic acid consumption were selected (Tables 3, 4) for vinification trials in Malbec wine at the final stage of alcoholic fermentation. Figure 6 shows the evolution of cell viability (log CFU/mL) and malic acid consumption (%MAC) of stored and selected samples, with UNQLp 11 fresh culture grown in MRS broth as a positive control, and wine without bacterial inoculation as a negative control.

All treatments showed a decrease in bacterial viability over time. Figure 6A shows that the sample with the lowest cell survival and lowest MAC (71%) was the one grown at pH 6 and dehydrated at 60 °C for 30 min. The sample with the best performance was grown in WP at pH 4.5 and dried at 45 °C for 35 min, with a final MAC of 80%.

UNQLp 11 grown in AP-based medium at pH 6.0 with and without water addition showed a lower viability decrease than cultures grown at pH 4.5 during the fermentation process. Also, samples grown in AP-based medium without water addition showed a lower viability decrease than those grown in the diluted medium (APW) (Figure 6B and Table 5). The highest MAC values after 20 days of incubation were 60 and 80% for samples grown in AP and APW media at pH 6.0, respectively (Figure 6B).

**Table 5:** Survival and kinetic parameters of malic acid consumption (Equation 3) of *Lpb. plantarum* UNQLp 11 after 20 days of inoculation in Malbec wine.

**Culture media**	**AP pH 4.5**	**AP pH 6.0**	**APW pH 4.5**	**APW pH 6.0**	**WP pH 4.5**	**WP pH 6.0**	**Fresh control**	**Negative control**
Drying condition	45 °C−35 min	45 °C−35 min	45 °C−35 min	45 °C−35 min	45 °C−35 min	60 °C−30 min	–	–
Rehydration treatment	MRS	MRS	MRS	MRS	PS	PS	–	–
No (CFU/mL)	2.5 × 10^5^	3.1 × 10^6^	6.67 × 10^5^	1.37 × 10^5^	6.0 × 10^5^	7.0 × 10^5^	1.30 × 10^7^	–
Log N/N_0_	−1.597	−0.927	−3.820	−2.662	−1.900	−2.694	−1.507	–
Final MAC (%)	50.78 ± 1.40	79.95 ± 2.35	55.21 ± 8.89	63.52 ± 8.13	80.53 ± 1.68	71.09 ± 4.35	90.64 ± 6.52	3.27 ± 6.56
*k*	0.770	0.254	0.271	0.060	0.574	0.156	0.110	0.134
*R* ^2^	0.90	0.98	0.83	0.91	0.99	0.98	0.930	0.31
% MAi	55.30	87.43	64.65	96.49	79.57	76.09	100	6.0

N_0_, number of viable cells at time = 0; Log N/N_0_, change in the number of viable cells after 20 days of incubation; Final MAC (%), percentage of malic acid consumed after 20 days of incubation; *k*, constant of first-order exponential decay; *R*^2^, coefficient of determination; %MA*i*, percentage of maximum malic acid consumption (time = infinite).

For cultures grown in APW, viability decreased under both preservation conditions compared to the fresh control. A loss of approximately 3 log units was observed for the culture at pH 6.0 (Figure 6C).

For both AP-based media, the condition in which cell viability was best maintained was with the initial culture at pH 6. It also appears that the dilution of AP in water negatively affected the stability of the culture in the vinification process.

## 4 Discussion

Malolactic starter cultures are valuable tools in winemaking for achieving a controlled and high-quality malolactic fermentation. The industrial application of these starter cultures largely depends on the preservation technologies used to ensure the long-term delivery of stable cultures in terms of viability and activity. The most used methods are freezing and freeze-drying, which reduce water activity, inhibiting deterioration reactions. Due to their high cost and time-consuming nature, alternative methods such as fluidized bed drying have also been explored. However, the impacts of the fluidized bed drying process on bacterial cultures and their subsequent functionality in wine have not been described. This dehydration method is used to preserve sensitive bioactive compounds, thereby achieving high viability after drying and subsequent storage of bacterial starter cultures. Additionally, it does not subject microorganisms to freeze-drying stress (freezing at −80 or −20 °C) and avoids heat damage, as is the case with spray drying (60 or 70 °C) (Strasser et al., 2009).

To analyze the effects of fluidized bed drying on *Lpb. plantarum* UNQLp 11, the strain was grown in two alternative culture media based on supplemented WP and AP, mixed with manioc starch, and dehydrated at 45 and 60 °C, with samples taken at different times (10, 20, 30, and 35 min). Water activity (a_w_) and moisture content of these samples were measured to study the decrease in a_w_ during the dehydration process and the desorption isotherms at both temperatures. Results showed that the decrease in a_w_ was faster at 60 °C.

Both temperature desorption isotherms exhibited a typical sigmoidal curve (Bell and Labuza, 2000) with a good fit to the GAB model (Figure 2B), as indicated by the determination coefficient (*R*^2^) of 0.99 (Table 2). By determining these isotherms, we aimed to optimize the drying conditions for bacterial preservation and thus contribute to the improvement of industrial processes that rely on the stability of microorganisms, such as in food applications. This provides essential insights into the water retention properties, which directly influence their stability during storage. According to our results, water activity is critical to understanding microbial behavior, as it impacts cell metabolism, stability, and survival, as can be seen in Figure 5, where samples with low a_w_ showed better survival after 1 year of storage.

In the GAB model, the monolayer moisture content (Mo) is defined as the amount of water that is strongly bound at all active sites in the solid adsorbent phase of the food, and is considered the value at which food is stable during storage (Moraes et al., 2008). It is a measure of the availability of active sites for water sorption by the material and usually decreases with the increase in temperature. In this study, Mo increased, with values of 4.095 at 45 °C and 4.862 at 60 °C treatment (Table 2). This behavior is not very common in food and can be explained by changes in the physical structure of the extrusion. When drying at 60 °C, the extruded material progressively breaks down, exposing new internal surfaces and functional groups. This structural disruption can increase the number of high-affinity adsorption sites available for water binding, increasing the solubility of solutes and leading to retention of more water molecules in the monolayer (Ribeiro et al., 2016). In contrast, the G value decreased as the temperature increased (Table 2). A higher G value at 45 °C could indicate stronger water binding in the monolayer (Quirijns et al., 2005). At higher drying temperatures (60 °C), the increased kinetic energy of water molecules reduced their binding strength to the matrix, which contributed to the lower G values observed. The *k* values also decreased with increasing temperature (Table 2), indicating that the binding energies associated with both mono- and multilayer sorption of water decline as temperature rises. Although drying at 60 °C can facilitate water desorption, some water molecules in the cell are structurally bound to membranes and proteins. The removal of this structural water can damage these macromolecules, potentially leading to cell death (Santivarangkna et al., 2008; Tymczyszyn et al., 2008).

The characterization of the drying process through the analysis of exponential decay curves and desorption isotherms allowed a clearer understanding of the moisture loss dynamics. The next step involved assessing the impact of these drying conditions on biological viability by comparing samples before and after dehydration at various time intervals and both drying temperatures. We also analyzed the behavior of the samples stored for 12 months.

The results showed that UNQLp 11 samples grown in WP- and AP-based media were highly susceptible to the damage induced by fluidized bed drying, particularly at high temperature (Figure 3), as compared to freeze-drying (Brizuela et al., 2021; Navarro et al., 2024), where cell loss is lower. This variation may be due to the intrinsic tolerance of the strain studied, as well as the individual conditions used in each step, from cell growth to storage, which can significantly influence culture viability. It should be noted that the literature on fluidized bed drying of LAB is limited and presents methodological inconsistencies, highlighting the need for more rigorous research to establish standardized protocols. Bensch et al. (2014) studied the effects of fluidized bed drying and storage on the viability of a *Lpb. plantarum* strain, and found that drying caused substantial damage to the cell membrane, confirmed by flow cytometry and plate counting, probably due to dehydration and oxidative stress. Strasser et al. (2009) and Wu et al. (2021) found that bacterial concentrates of LAB were more sensitive to fluidized bed bottom-spray drying than to freeze-drying, but with higher cell viability than in this study. However, fluidized bed bottom-spray drying is different from the drying conditions used in this study, where LAB strains could be microencapsulated with glucose, trehalose, maltodextrin, among other protective agents.

This evidence suggests that in future experiments, we could use other compounds such as trehalose, maltodextrin, and different oligosaccharides that have been reported to significantly improve the stability of lactic acid bacteria during drying processes, mainly by stabilizing membranes and proteins under thermal and osmotic stress (Strasser et al., 2009; Santivarangkna et al., 2008).

Moreover, optimization of drying temperature should also be considered. Previous reports have shown that moderate reductions in temperature and adjustments in exposure times can limit thermal and osmotic damage in lactic acid bacteria during fluidized bed drying and related dehydration processes (Türker et al., 2006; Wu et al., 2021). Although we evaluated two temperatures and several drying times, a more exhaustive investigation of these variables (for example drying at 30–40 °C) could help identify milder conditions compatible with higher cell survival.

In this study, the bacterial samples were mixed with manioc starch before the drying process, which could have a cellular protective effect. Abadias et al. (2001) reported that starch and cellulose offer protection to microbial cells during the freeze-drying process in *Lactobacilli*. Starch not only granulizes cells, a necessary step for fluidized bed drying, but also provides a microenvironment that protects cells from unfavorable conditions, such as oxygen and light exposure during drying and/or storage. We estimate that the protective effect depends on the starch granule and the bacterial species subjected to drying.

Furthermore, this study analyzed the fluidized bed drying of UNQLp 11 grown in two AP-based media (AP and APW), which could have a protective effect on this strain. Several studies have shown that the chemical compounds in AP, such as pectin and polyphenols, make this by-product suitable for valorization as a potential prebiotic (Calvete-Torre et al., 2021). In the study by Vlad et al. (2022), the authors demonstrated that the high polyphenolic content and antioxidant activity of the powder with ethanolic extract of apple pulp supplemented with MRS allowed improving the viable cell count of a probiotic bacterial species, *Loigolactobacillus bifermentans*.

Viability loss (log N/N_0_) of UNQLp 11 after 12 months of storage was also analyzed as a function of a_w_ after fluidized bed drying at both temperatures, regardless of the growth conditions. The optimal a_w_ range was between 0.22 and 0.30 at 45 °C, indicating that lower temperatures caused less cell damage. Tonon et al. (2009) showed that an a_w_ below 0.3 had a positive effect on powder consistency, decreasing biochemical reactions and therefore prolonging the shelf life of products. Poddar et al. (2021) analyzed the fluidized bed drying and storage of *Lactobacillus paracasei* with different water activities and at different temperatures; the results demonstrated that higher storage temperature and water activity of the powders lead to increased bacterial death, irrespective of the drying matrices used. The bacterial powders stored at 4 °C with a_w_ 0.3 had great bacterial viability.

As already mentioned, for the selection of new enological microorganisms, it is essential that the selected strain remains technologically active after drying and storage. In our previous studies, the enological strain UNQLp 11 was grown in alternative media, dried by freeze-drying, and technologically evaluated in synthetic wine. In a study conducted by Brizuela et al. (2021), UNQLp 11 was grown in a WP-based medium, freeze-dried, and stored at different temperatures for approximately 2 months. In this case, UNQLp 11 remained viable during laboratory-scale winemaking, and %MAC varied depending on the storage time and drying conditions. In the study by Navarro et al. (2024), UNQLp 11 was grown in an AP-based medium, freeze-dried, and subsequently analyzed for viability and %MAC in synthetic wine. This medium yielded bacterial biomass values similar to those obtained with the commercial medium MRS, and the %MAC values were not modified by the drying process.

In the present study, UNQLp 11 was grown in WP-based media, dehydrated by fluidized bed drying, stored vacuum-sealed for 1 year at 4 °C without light exposure. It was then rehydrated, and its viability and technological functionality in synthetic wine were evaluated. Viability results were heterogeneous as they varied depending on the pH, growth medium, and drying times. As expected, the %MAC in synthetic wine depended on the ability of cells to survive. Under all conditions, the UNQLp 11 strain remained culturable in synthetic wine after 7 days of inoculation, confirming its ability to adapt to a stressful environment similar to that of wine (high ethanol concentration and low pH) after drying, storage, and rehydration.

UNQLp 11 grown in WP-based medium and rehydrated with physiological solution showed the best technological performance with respect to %MAC in laboratory-scale fermentation of synthetic wine, ranging from 75 to 100%. However, selected AP culture samples rehydrated with physiological solution after 1 year of storage showed low %MAC during the week of incubation, ranging from 18 to 40%. In contrast, rehydration with MRS broth showed values between 75 and 100%. The latter reflects the need for nutrient supplementation for cell repair after the drying process (Balmaseda et al., 2021; Brizuela et al., 2021). However, it is important to note that this practice is not uncommon in the starter culture industry, where many freeze-dried or spray-dried products are supplied with instructions for rehydration in nutrient-enriched media to ensure optimal recovery of viability and technological performance. For instance, companies such as Chr. Hansen, Lallemand, and DSM offer starter cultures that require reactivation in skim milk or media supplemented with yeast extract, peptones, or other nitrogen sources. In particular, Lallemand's 1-STEP^®^ malolactic fermentation cultures involve a brief nutrient-supplemented rehydration step to guarantee robust activity under winemaking conditions. These examples demonstrate that nutrient supplementation during rehydration is a recognized and widely applied industrial strategy. Moreover, we could consider to prove other rehydration medium by incorporating cost-effective supplements, such as yeast extract from beer lees (Brizuela et al., 2025), peptones, or whey protein (Balmaseda et al., 2021).

Malic acid decarboxylation capacity and cell survival were also analyzed in Malbec wine at the final stage of alcoholic fermentation. AP and APW cultures with an initial pH of 6.0 and dried at 45 °C for 35 min exhibited the best stability over the incubation period in Malbec wine, with lower loss of viability and higher %MAC than cultures with an initial pH of 4.5. UNQLp 11 grown in WP-based media showed better stability and a higher %MAC when the initial pH culture was 4.5 and when dried at 45 °C for 35 min. The difference in behavior between the AP and WP cultures under varying pH conditions could have significant implications for designing fermentation processes in lactic biomass production (Guyot et al., 2000; Ren et al., 2022), particularly regarding the manipulation of pH as a tool to optimize and ensure bacterial culture stability.

Finally, the preservation of LAB starter cultures by alternative drying processes has attracted increasing attention, due to the high costs and energy consumption of freezing and freeze-drying (Santivarangkna et al., 2007). Fluidized bed drying offers several potential advantages compared to freeze-drying: (i)Significantly lower operating costs, as fluidized bed drying relies on hot air at moderate temperatures, whereas freeze-drying requires highly specialized equipment, long processing times, and considerable energy consumption (Wu et al., 2021), (ii) continuous and scalable processing, since fluidized bed drying is already widely used in the food and pharmaceutical industries for powders and granules, facilitating industrial adoption without the need for highly specialized infrastructure, (iii) shorter processing times, which enhance productivity and may help reduce the degradation of sensitive bioactive compounds, and (iv) lower energy consumption and greater environmental sustainability, as fluidized bed drying does not require intensive vacuum and refrigeration conditions as in freeze-drying, making it a more energy-efficient option with a smaller carbon footprint (Santivarangkna et al., 2008). Nonetheless, we also acknowledge certain limitations: cell viability losses are typically higher compared to freeze-drying, and further optimization of formulation parameters (aforementioned; protectants and drying conditions) is needed to achieve comparable survival rates.

Therefore, optimizing sustainable bacterial preservation processes in the food industry requires an integrated approach that ensures the viability and functionality of microorganisms while promoting a more efficient and responsible production.

## 5 Conclusion

LAB preservation techniques are critical for wine commercialization. The results of this study demonstrate that fluidized bed drying is a promising method for the preservation of the *Lactiplantibacillus plantarum* UNQLp 11 strain, with potential for application in the wine industry. Although cell viability was more severely affected compared to samples dried with freeze-drying, the strain maintained its technological functionality, i.e., its ability to survive and consume malic acid in synthetic wine and Malbec wine, significantly shortening the process times. This study establishes a foundation for understanding the water-retention properties of the dehydrated bacterial strain. Although these results provide an initial insight into the behavior of a fluidized bed dried winemaking strain, they improve our understanding of its performance in future drying trials and pilot-scale drying. Rehydration with a nutrient medium, such as MRS broth, significantly improved the technological functionality of the samples grown on apple pomace. This finding indicates that nutritional supplementation can aid cell recovery after the stress of drying and storage. Fluidized bed drying is a viable and sustainable alternative, but its successful application in the industry requires a comprehensive approach to ensure the stability and functionality of bacterial cultures.

## Data Availability

The raw data supporting the conclusions of this article will be made available by the authors, without undue reservation.
